# Serum concentrations of vascular endothelial growth factor and nitrite as an estimate of in vivo nitric oxide in patients with gastric cancer

**DOI:** 10.1038/sj.bjc.6690573

**Published:** 1999-07

**Authors:** A Eroğlu, S Demirci, A Ayyildiz, H Kocaoğlu, H Akbulut, H Akgül, H A Elhan

**Affiliations:** Departments of Surgical Oncology, Biochemistry, Medical Oncology and Biostatistics, Ankara University Medical School, 06100, Ankara, Turkey

**Keywords:** gastric cancer, nitric oxide, VEGF, serum

## Abstract

The importance of tumour angiogenesis in the process of tumour growth and progression in solid tumours has been widely accepted. Among many angiogenic factors, vascular endothelial growth factor (VEGF) has been shown to play a major role in the development and dissemination of the malignant tumours. Nitric oxide (NO) production was also observed in solid tumour tissues. NO has been reported to play an important role for the mitogenic effect of VEGF in the angiogenic process. However, little is known about the correlation between VEGF and NO in circulating levels. Therefore, we investigated serum VEGF and NO concentrations in human gastric cancers as well as healthy individuals, and examined the influence of tumour stage on circulating level of VEGF. The study consisted of 11 healthy individuals and 37 patients with primary gastric cancer who did not receive any prior therapy. Patients were categorized into four groups according to TNM classification. The level of VEGF_165_ in preoperative sera of gastric cancer patients and healthy donors was assayed using the quantitative sandwich enzyme immunoassay technique. NO concentration was estimated indirectly from serum nitrite. The ANOVA test showed a significant difference in serum VEGF_165_ concentrations between tumour stages (*P* < 0.001). A striking relationship was found between serum NO levels and tumour stage (*P* < 0.001). A significant difference was also seen between healthy individuals and patients with stage 1 disease. The present study suggested that large tumour burden was associated with significantly increased levels of VEGF_165_ and NO. © 1999 Cancer Research Campaign


*British Journal of Cancer* (1999) **80**(10), 1630–1634
© 1999 Cancer Research Campaign
Article no. bjoc.1999.0573

### Serum concentrations of vascular endothelial growth factor and nitrite as an estimate of in vivo nitric oxide in patients with gastric cancer

**A Eroğlu[1], S Demirci[1], A Ayyildiz[2], H Kocaoğlu[1], H Akbulut[3], H Akgül[1] and HA Elhan[4]**

Departments of [1]Surgical Oncology, [2]Biochemistry, [3]Medical Oncology and [4]Biostatistics, Ankara University Medical School, 06100, Ankara, Turkey

**Summary** The importance of tumour angiogenesis in the process of tumour growth and progression in solid tumours has been widely accepted. Among many angiogenic factors, vascular endothelial growth factor (VEGF) has been shown to play a major role in the development and dissemination of the malignant tumours. Nitric oxide (NO) production was also observed in solid tumour tissues. NO has been reported to play an important role for the mitogenic effect of VEGF in the angiogenic process. However, little is known about the correlation between VEGF and NO in circulating levels. Therefore, we investigated serum VEGF and NO concentrations in human gastric cancers as well as healthy individuals, and examined the influence of tumour stage on circulating level of VEGF. The study consisted of 11 healthy individuals and 37 patients with primary gastric cancer who did not receive any prior therapy. Patients were categorized into four groups according to TNM classification. The level of $VEGF_{165}$ in preoperative sera of gastric cancer patients and healthy donors was assayed using the quantitative sandwich enzyme immunoassay technique. NO concentration was estimated indirectly from serum nitrite. The ANOVA test showed a significant difference in serum $VEGF_{165}$ concentrations between tumour stages ($P < 0.001$). A striking relationship was found between serum NO levels and tumour stage ($P < 0.001$). A significant difference was also seen between healthy individuals and patients with stage 1 disease. The present study suggested that large tumour burden was associated with significantly increased levels of $VEGF_{165}$ and NO.

**Keywords:** gastric cancer; nitric oxide; VEGF; serum

Tumour angiogenesis is required for the growth of solid tumours and metastasis. The significance of solid tumour angiogenesis as a prognostic factor has been investigated in many human tumours including gastric carcinoma (Maeda et al, 1995; Araya et al, 1997). Among the various angiogenic factors, vascular endothelial growth factor (VEGF) has been regarded as the most likely candidate for the induction of angiogenesis in tumour growth (Senger et al, 1993).

Nitric oxide (NO) is a biologically active mediator derived from L-arginine, which is catalysed by NO synthase (NOS) (Moncada et al, 1991). NO produces multiple effects that can influence the outcome of metastasis and regulates vasodilatation which affect tumour cell arrest in capillaries (Dong et al, 1994). In recent years, some studies have shown that NOS is present in human tumour tissues, including gynaecological cancer (Thomsen et al, 1994), breast cancer (Thomsen et al, 1995), brain tumour (Cobbs et al, 1995), gastric cancer (Miles et al, 1996) and head and neck cancer (Gallo et al, 1998).

Although many studies have shown that VEGF was over-expressed by a variety of human cancer tissue, relatively little is known about the behaviour of VEGF in the circulating level in patients with gastric cancer. Moreover, previous investigations have reported that several murine and human tumour cell lines produce NO. However, still little is about the correlation between the NO and VEGF in the circulating levels.

The aim of the present study was to investigate serum concentrations of VEGF and nitrite as an estimate of in vivo NO in patients with gastric cancer as well as healthy individuals and to examine the influence of tumour stage and tumour grade on circulating levels of NO and VEGF.

### MATERIALS AND METHODS

### Study populations

The study consisted of 11 healthy individuals without any evidence of disease (e.g. liver dysfunction, diabetes etc.) and 37 patients with primary gastric carcinoma who were treated at our department. No patient had received chemotherapy or radiation therapy before surgery. None of the subjects had renal dysfunction, infection, or gastrointestinal bleeding. In addition, none of them received any antibiotics or vasoactive drugs before the sera were obtained.

The patients ranged in age from 32 to 81 years (mean age 61 years). There were 21 men and 16 women. To investigate whether NO and VEGF concentrations in serum could reflect the tumour stage, we classified the patients according to 1997 TNM classification (Sobin and Wittekind, 1997), and measured the VEGF and estimated NO concentrations in their serum. In addition, patients were categorized into three groups according to tumour grade. Patients' characteristics are summarized in Table 1.

*Received 22 September 1998*
*Revised 12 January 1999*
*Accepted 27 January 1999*

*Correspondence to:* A Eroğlu, Ragip Tüzün cad., 430-2, Y. Mahalle, 06180, Ankara, Turkey

**Table 1** Characteristics of 37 patients with gastric cancer

| Characteristics | No. of patients |
|---|---|
| Mean age (range) | 61 years (32–81) |
| Sex | |
| Male | 21 |
| Female | 16 |
| Tumour stage | |
| 1 | 4 |
| 2 | 8 |
| 3 | 15 |
| 4 | 10 |
| Tumour grade (G) | |
| G1 | 6 |
| G2 | 12 |
| G3 | 19 |
| Tumour location | |
| Upper-third stomach | 6 |
| Middle-third stomach | 9 |
| Lower-third stomach | 22 |

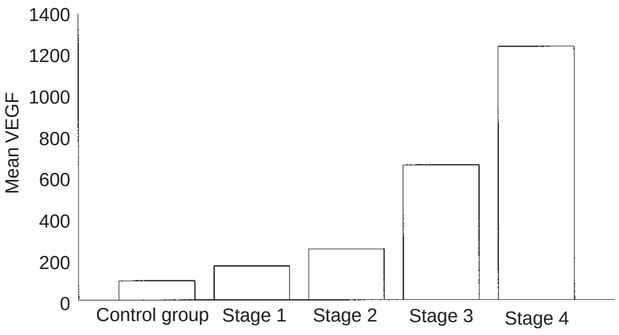

**Figure 1** Serum VEGF-165 levels (pg $ml^{-1}$) in control subjects and gastric cancer patients

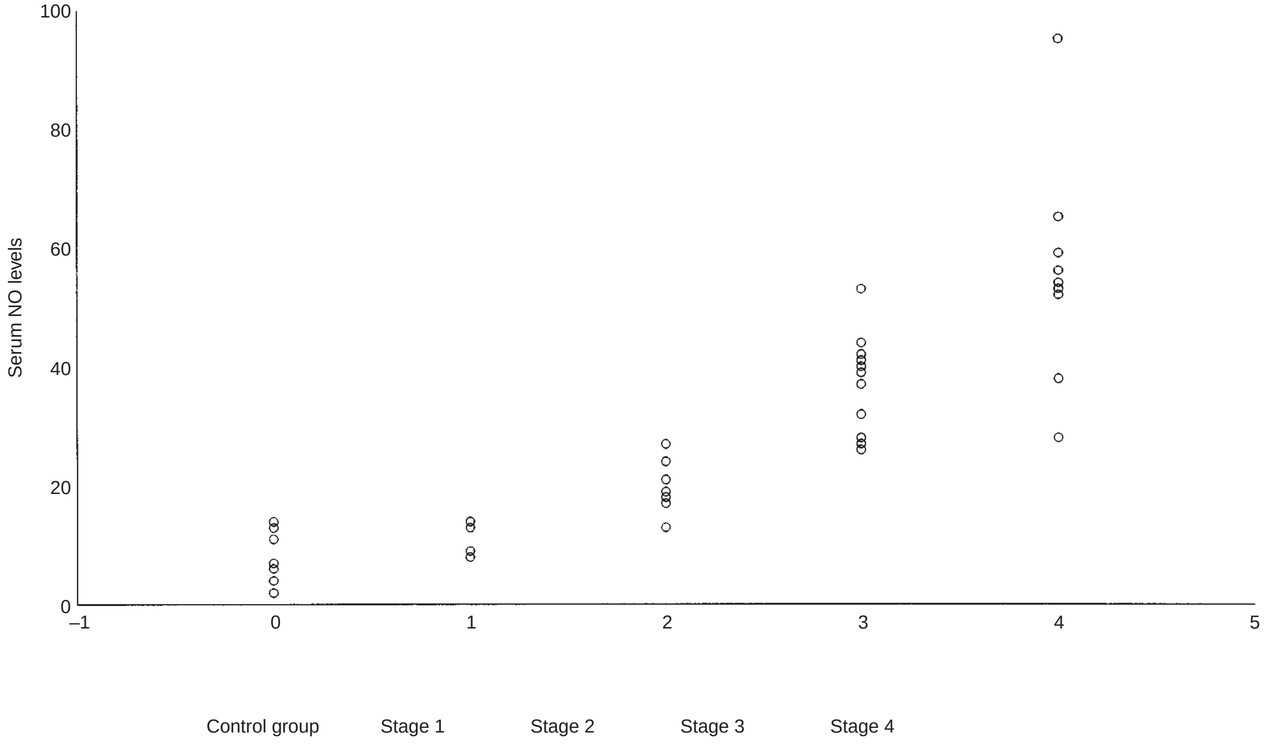

**Figure 2** Serum NO levels in control group and gastric cancer patients

### Laboratory assay

Sera were obtained from patients who had not undergone surgery. All patients and control subjects fasted for at least 12 h. Venous blood samples were drawn into a tube and allowed to clot for 20 min before centrifugation at 3000 rpm for 10 min. The serum samples were stored at –80°C until analysis. The VEGF levels in preoperative sera of patients and healthy donors were measured by using the quantitative sandwich immunoassay technique (Quantikine, R&D Systems, UK).

Griess reaction was the method of choice to evaluate serum nitrite/nitrate levels with the following modifications. In vivo NO concentration* estimation was done via analysis of nitrites ($NO^{-2}$) in serum without reduction of nitrates ($NO^{-3}$) step, as it has recently been suggested that inclusion of nitrates in the determination of in vivo NO* causes an overestimation of approximately 500% than when NO was measured by the haemoglobin reaction (Privat et al, 1997). Briefly, serum samples were deproteinized with 0.6 N perchloric acid and the supernatants were assayed for nitrite concentration through recording the absorbance at 540 nm.

$$NO_{\text{in vivo}} = (OD_{\text{serum}} - OD_{\text{serum blank w / H20}}) \times F\ \mu M$$

F = 100 (conversion factor from $A_{540}$ to µM nitrite from standard curve) due to the equations valid for oxygenated solutions (1)–(3) (Hevel and Marletta, 1994).

*© 1999 Cancer Research Campaign*

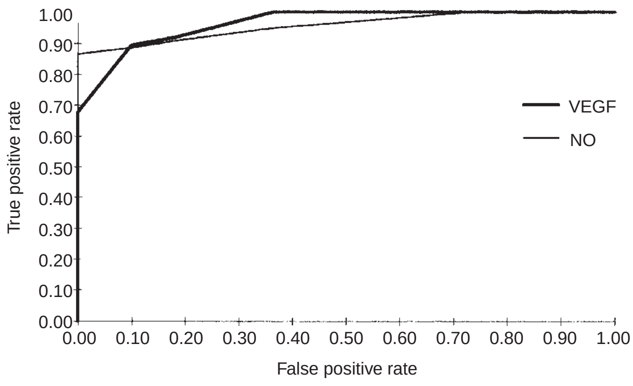

**Figure 3** ROC curves of VEGF and NO. The sensitivity and the specificity for VEGF and NO are computed as 91.9% and 81.8%; 89.2% and 90.9% respectively

$$2NO^* + O_2 \rightarrow 2NO_2^* \quad (1)$$
$$NO^* + NO_2^* \rightarrow N_2O_3 \rightarrow 2\ NO_2^- \quad (2)$$
$$+2NO_2^* \rightarrow N_2O_4 + H_2O \rightarrow NO_2^- + NO_3^- \quad (3)$$
$$3NO^* \rightarrow 3\ NO_2^-$$

The NO and VEGF values for each serum sample were obtained from duplicate assays.

### Statistical analysis

Results were expressed as the mean ± standard deviation (s.d.). Comparison of VEGF and NO levels between the groups was evaluated by analysis of variance (ANOVA) test. The relationships between the NO and VEGF levels were reported as Spearman product-moment correlation coefficient ($r_s$). Cut-off value for VEGF and NO was calculated by using receiver operating characteristic (ROC) analysis. Areas under the ROC curves of VEGF and NO were also compared. For all tests, a *P*-value less than 0.05 was accepted as significant.

The statistical analysis was performed by Statistical Package for Social Sciences (SPSS for MS Windows Release 5.0, Chicago, IL, USA).

### RESULTS

Two major histological types considered in this study were adenocarcinoma, diagnosed in 28 patients, and mucinous carcinoma, defined as when a mucinous component was evident in more than 50% of the tumour section in nine patients.

Tumours were staged by TNM classification. Accordingly, there were four patients in stage 1 (T1 N0 M0 in one, T1 N1 M0 in two, T2 N0 M0 in one), eight in stage 2 (T2 N1 M0 in four, T3 N0 M0 in four), 15 in stage 3 (T3 N1 M0 in six, T3 N2 M0 in eight, T4 N0 M0 in one) and ten in stage 4 (T3 N3 M0 in four, T4 N2 M0 in two, T4 N2 M1 in two, T4 N3 M1 in two). The distribution of patients according to the degree of histological differentiation of the tumour was as follows: well-differentiated tumours (G1) in six patients, moderately differentiated (G2) in 12, poorly differentiated tumours (G3) in 19 patients.

No significant correlation was seen between histological type of the tumour and serum VEGF concentrations. Moderately differentiated and poorly differentiated tumours were related to an increased mean level of VEGF, 695.2 ± 311.4 pg $ml^{-1}$ and 759.3 ± 400.5 pg $ml^{-1}$ respectively, compared with well-differentiated tumours (349.1 ± 247.3 pg $ml^{-1}$), but these differences were not found as statistically significant ($P > 0.05$). Also, there was no significant difference between men and women and between tumour locations ($P > 0.05$).

The distribution of the mean level of VEGF on the basis tumour stage was as follows: 166 ± 52.4 pg $ml^{-1}$ (range 122.6–242.3) in stage 1; 248.1 ± 107.7 pg $ml^{-1}$ (range 168.2–504) in stage 2; 658.6 ± 324 pg $ml^{-1}$ (range 209.6–1273) in stage 3; and 1233.6 ± 317.3 pg $ml^{-1}$ (range 798.7–1777) in stage 4. It was seen that large tumour burden was associated with increased level of VEGF. The mean level of VEGF in sera from healthy individuals was 94.5 ± 55.7 pg $ml^{-1}$ (range 36.1–218). A statistically significant difference in serum VEGF concentrations was found between the groups ($P < 0.001$) (Figure 1).

As shown in Figure 2, serum NO levels in patients with stage 1 gastric carcinoma (mean 11 ± 2.9 μM, range 8–14) were significantly higher than those in healthy individuals (mean 8.5 ± 4.3 μM, range 2–11).

The ANOVA test also showed a significant difference in serum NO concentrations among stage 1, stage 2 (mean 20.4 ± 4.5 μM, range 13–27), stage 3 (mean 36.8 ± 7.4 μM, range 26–53) and stage 4 disease (mean 55.6 ± 17.5 μM, range 38–95) ($P < 0.001$). On the other hand, the serum nitrite level was higher in grade 3 tumour patients (mean 40 ± 14.8 μM) compared with grade 1 (mean 26.6 ± 10.8 μM) or grade 2 tumour patients (mean 32 ± 24.3 μM). But, these differences were not statistically significant ($P > 0.05$). Moreover, there was no significant difference with respect to patient gender and tumour location. In addition, when we correlated serum VEGF level with serum NO concentration, we found that the increased VEGF level was statistically associated with an elevated nitrite level ($r_s = 0.8$ and $P < 0.0001$).

By ROC analysis, the sensitivity and the specificity for VEGF and NO were computed as 91.9% and 81.8%; and 89.2% and 90.9% respectively. The cut-off levels of serum VEGF and NO concentrations were found to be 149.9 pg $ml^{-1}$ and 13 μM respectively. Area under the curves of VEGF and NO was computed as 0.9619 ± 0.026 and 0.9582 ± 0.0257 respectively. There was no statistical differences between the area under two curves (Figure 3).

### DISCUSSION

The importance of tumour angiogenesis in the process of tumour growth and progression in solid tumour has been widely accepted (Fidler and Ellis, 1994). VEGF, also known as vascular permeability factor (VPF), is a 34–42 kDa multifunctional cytokine that is an important regulator of angiogenesis. VEGF has been shown to play an important role in the development and metastasis of the solid tumours (Senger et al, 1993).

Previous studies have demonstrated that VEGF was overexpressed by a variety of human cancer tissues. In recent years, some authors have also reported on the relationship between tumour angiogenesis and prognosis in patients with gastric carcinoma, both disease-free and overall survival rates decrease with increasing microvessel count (Maeda et al, 1995; Araya et al, 1997). Moreover, multivariate analysis indicated that the overexpression of VEGF is an independent prognostic factor in gastric cancer patients (Maeda et al, 1996). However, relatively little is known about the circulating level of VEGF. A recent study has

© 1999 Cancer Research Campaign shown that serum VEGF level was significantly elevated in some population of cancer patients including those of gastric cancer compared with control subjects (Yamamoto et al, 1996).

In the present study, we determinated serum VEGF concentrations in patients with gastric carcinoma as well as healthy individuals. Here we reported our findings from serum samples from different tumour stages of human gastric cancer. Elevated serum levels of VEGF were found in advanced disease patients when compared to early-stage disease and healthy individuals. This suggests that high VEGF level is related to advanced tumour stage. Although the number of patients studied was small, we suggest that VEGF assay may be useful in predicting the advanced gastric carcinoma.

Important steps in angiogenesis include invasion of the vascular wall of the parent vessel by activated endothelial cells which then proliferate, migrate and form the lumen of the new vessel (Fidler and Ellis, 1994). The role of NO in tumour angiogenesis is not sufficiently understood. Although NO was first identified in endothelial cells, it is well known to be generated by a variety of solid human tumours.

Previous investigations have shown NO to cause increased tumour blood flow, oedema and vascular permeability (Nathan and Xie, 1994; Fukumura et al, 1997). NO production by tumour cells may influence tumour growth and metastasis (Pipili-Synetos et al, 1993; Jenkins et al, 1995). The functions of NO in tumours potentially facilitate tumour growth. Moreover, tumour blood flow and vessel diameter of experimental tumours in mice decreased after the treatment with NOS inhibitors (Andrade et al, 1992; Thomsen et al, 1997).

NOS activity was observed in solid human tumour tissues, including gynaecological cancer (Thomsen et al, 1994), breast cancer (Thomsen et al, 1995), brain tumour (Cobbs et al, 1995), gastric cancer (Miles et al, 1996) and head and neck cancer (Gallo et al, 1998). Furthermore, this enzyme activity in the tumour tissues correlated with grade.

In a recent study, it was suggested that the increased plasma nitrite/nitrate levels were correlated to tumour volume in patients with hepatocellular carcinoma (Moriyama et al, 1997). In the present study, we have estimated serum NO as nitrite in gastric cancer patients and control subjects. We have investigated the effect of tumour stage on the production of NO in sera of gastric cancer patients. A striking relationship was found between serum NO levels and tumour stage ($P < 0.001$). Although the serum NO level was elevated in high-grade tumour compared with low-grade tumour, this difference was found to be statistically insignificant. The slightly lower serum NO concentrations were observed in stage I disease; however, the highest serum level of NO was found in stage 4 tumour. Serum NO levels were higher in patients with advanced gastric cancer and were also positively correlated with increased VEGF levels.

Recently, Gallo et al (1998) reported an interesting analysis of NO production and angiogenesis in 27 patients with head and neck cancer. Their results revealed a strong positive correlation between the expression of NOS and tumour angiogenesis and tumour progression. To our knowledge, there has been no report about the relationship between NO and VEGF concentration in sera in gastric cancer patients. We therefore examined whether serum NO levels correlate with serum VEGF levels.

In this study, we show that an increase in NO levels is found in gastric cancer, is associated with elevated VEGF levels and correlates with tumour stage. This positive correlation between tumour stage and serum NO and VEGF levels in patients with gastric cancer suggests that NO might play a role in the biology of the gastric cancer. The relationship observed between tumour stage, VEGF and NO may be due to ongoing neovascularization associated with increasing tumour burden. It needs further studies to elucidate a precise role for the NO pathway in tumour angiogenesis. In addition, antiangiogenetic therapy has been investigated and may be a new treatment for metastatic disease in the future (Folkman, 1997). Accordingly, it is also recommended that the monitoring of VEGF and NO levels in the circulation may be useful to assess the anti-angiogenetic therapy.

### REFERENCES

Andrade SP, Hart IR and Piper PJ (1992) Inhibitors of nitric oxide synthase selectively reduce flow in tumour-associated neovasculature. *Br J Pharmacol* **107**: 1092–1095

Araya M, Terashima M, Takagane A, Abe K, Nishizuka S, Yonezawa H, Irinoda T, Nakaya T and Saito K (1997) Microvessel count predicts metastasis and prognosis in patients with gastric cancer. *J Surg Oncol* **65**: 232–236

Cobbs CS, Brenman JE, Aldape KD, Bredt DS and Israel MA (1995) Expression of nitric oxide synthase in human central nervous system tumors. *Cancer Res* **55**: 727–730

Dong Z, Staroselsky AH, Qi X, Xie K and Fidler IJ (1994) Inverse correlation between inducible nitric oxide synthase activity and production of metastasis in K-1735 murine melanoma cells. *Cancer Res* **54**: 789–793

Fidler IJ and Ellis LM (1994) The implications of angiogenesis for the biology and therapy of cancer metastasis. *Cell* **79**: 185–188

Folkman J (1997) Antiangiogenic therapy. In: *Cancer Principles and Practice of Oncology*, DeVita VT Jr, Hellman S and Rosenberg SA (eds), pp. 3075–3085. Lippincott-Raven: Philadelphia

Fukumura D, Yuan F, Endo M and Jain RK (1997) Role of nitric oxide in tumor microcirculation. Blood flow, vascular permeability, and leukocyte-endothelial interactions. *Am J Pathol* **150**: 713–725

Gallo O, Masini E, Morbidelli L, Franchi A, Fini-Storchi I, Vergari WA and Ziche M (1998) Role of nitric oxide in angiogenesis and tumor progression in head and neck cancer. *J Natl Cancer Inst* **90**: 587–596

Hevel JM and Marletta MA (1994) Nitric oxide synthase assays. *Methods Enzymol* **233**: 250–258

Jenkins DC, Charles IG, Thomsen LL, Moss DW, Holmes LS, Baylis SA, Rhodes P, Westmore K, Emson PC and Moncada S (1995) Roles of nitric oxide in tumor growth. *Proc Natl Acad Sci USA* **92**: 4392–4396

Maeda K, Chung YS, Takatsuka S, Ogawa Y, Onoda N, Sawada T, Kato Y, Nitta A, Arimoto Y, Kondo Y and Sowa M (1995) Tumour angiogenesis and tumour cell proliferation as prognostic indicators in gastric carcinoma. *Br J Cancer* **72**: 319–323

Maeda K, Chung Y, Ogawa Y, Takatsuka S, Kang SM, Ogawa M, Sawada T and Sowa M (1996) Prognostic value of vascular endothelial growth factor expression in gastric carcinoma. *Cancer* **77**: 858–863

Miles DW, Happerfield LC, Thomsen LL and Filipe I (1996) Nitric oxide synthase activity and localization in gastric carcinoma. *Proc Am Assoc Cancer Res* **37**: 155

Moncada S, Palmer RM and Higgs EA (1991) Nitric oxide: physiology, pathophysiology, and pharmacology. *Pharmacol Rev* **43**: 109–142

Moriyama A, Masumoto A, Nanri H, Tabaru A, Unoki H, Imoto I, Ikeda M and Otsuki M (1997) High plasma concentrations of nitrite/nitrate in patients with hepatocellular carcinoma. *Am J Gastroenterol* **92**: 1520–1523

Nathan C and Xie QW (1994) Nitric oxide synthases: roles, tolls, and controls. *Cell* **78**: 915–918

Pipili-Synetos E, Sakkoula E and Maragoudakis ME (1993) Nitric oxide is involved in the regulation of angiogenesis. *Br J Pharmacol* **108**: 855–857

Privat C, Lantoine F, Bedioui F, van Brussel ME, Devynck J and Devynck MA (1997) Nitric oxide production by endothelial cells: comparison of three methods of quantification. *Life Sci* **61**: 1193–1202

Senger DR, Van De Water L, Brown LF, Nagy JA, Yeo KT, Yeo TK, Berse B, Jackman RW, Dvorak AM and Dvorak HF (1993) *Cancer Metastas Rev* **12**: 303–324

Sobin LH and Wittekind Ch (eds) (1997) *TNM Classification of Malignant Tumours,* pp 59–62. Wiley-Liss: New York

Thomsen LL, Lawton FG, Knowles RG, Beesley JE, Riveros-Moreno V and Moncada S (1994) Nitric oxide synthase activity in human gynecological cancer. *Cancer Res* **54**: 1352–1354

© 1999 Cancer Research Campaign

Thomsen LL, Miles DW, Happerfield L, Bobrow LG, Knowles RG and Moncada S (1995) Nirtic oxide synthase activity in human breast cancer. *Br J Cancer* **72**: 41–44

Thomsen LL, Scott JMJ, Topley P, Knowles RG, Keerie AJ and Frend AJ (1997) Selective inhibition of inducible nitric oxide synthase inhibits tumor growth in vivo: studies with 1400W, a novel inhibitor. *Cancer Res* **57**: 3300–3304

Yamamoto Y, Toi M, Kondo S, Matsumoto T, Suzuki H, Kitamura M, Tsuruta K, Taniguchi T, Okamoto A, Mori T, Yoshida M, Ikeda T and Tominaga T (1996) Concentrations of vascular endothelial growth factor in the sera of normal controls and cancer patients. *Clin Cancer Res* **2**: 821–826

*© 1999 Cancer Research Campaign*
